# Anesthesia management of a morbidly obese patient in prone position for lumbar spine surgery

**DOI:** 10.4103/0974-8237.65483

**Published:** 2010

**Authors:** Vaibhavi Baxi, Shashank Budhakar

**Affiliations:** Lilavati Hospital and Research Centre, Mumbai, India

**Keywords:** Anesthesia management, lumbar spine, morbid obesity, prone position

## Abstract

A morbidly obese, 45-year-old woman with a body mass index of 47 kg/m^2^ , presented with a prolapsed intervertebral disc of the lumbar spine for decompression and fixation. Anesthesia and surgical positioning of morbidly obese patient carries 3 main hazards, namely, morbid obesity, prone position, and airway preservation problems. Morbid obesity has its own hazards of deep vein thrombosis and pulmonary embolus. Here we describe anesthetic management, successfully dealing with the specific problems of this patient due to obesity.

## INTRODUCTION

Morbid obesity is an important health problem with constantly increasing incidence. It leads to coronary artery disease, hypertension, dyslipidemia, diabetes mellitus, gallbladder disease, degenerative joint disease, obstructive sleep apnea, and socioeconomic and psychosocial problems.[1] The outcomes of these problems are closely associated with body mass index (BMI). A BMI of 25 kg/m^2^ and below is considered as normal, 25-30 kg/m^2^ as at low risk, above 40 kg/m^2^ as at high risk, and above 50 kg/m^2^ is considered as serious morbid obesity.[2] Prone positioning of patients during anesthesia is associated with predictable changes in the physiology but also with a number of complications, and safe use of the prone position in morbidly obese patients requires an understanding of both.

Preoperative optimization is very important for the success of anesthetic intervention and safety of morbidly obese patients. We wish to elaborate our perioperative experience in a morbidly obese patient (BMI = 47 kg/m^2^ ) in prone position.

## CASE REPORT

A 45-year-old woman weighing 122 kg with a height of 161 cm (BMI = 47 kg/m^2^ ) was scheduled to undergo decompression and fixation of lumbar spine. Preoperative evaluation revealed that she was breathless in supine position and was used to sleeping in a recumbent position. She had hypertension, diabetes mellitus, as well as limited effort capacity because of her obesity. Her blood pressure and blood glucose levels were within normal limits with antihypertensive agents (.amlodepine) and oral hypoglycemic drugs (glimipride). She gave history of snoring and symptoms of obstructive sleep apnea. Her heart rate was 88 beats/min and blood pressure in recumbent position was 140/90 mmHg. On auscultation, heart sounds were normal. Air entry was bilaterally decreased at the bases with a respiratory rate of 24–26/min at rest with saturation of peripheral oxygen (SpO_2_ ) of 93% on air. Her Mallampati score was class 3, mouth opening was adequate, and she had a short neck (thyromental distance was 6 cm) and a thick pad of fat all around her neck with a neck circumference of 42 cm. Routine biochemical results were within normal limits. The electrocardiogram showed a sinus rhythm. Increased bronchovascular markings were seen in the preoperative chest radiograph. Preoperative echocardiogram showed a concentric hypertrophy of the left ventricle and a borderline-sized left atrium. *Left ventricular ejection fraction* was 50% with no regional wall motion abnormality. Pulmonary artery systolic pressure was 72 mmHg and tricuspid regurgitation was GrI/III. Analysis of blood gases on room air (fraction of inspired oxygen 0.21) revealed partial pressure of oxygen (PaO_2_ ) of 65 mmHg, partial pressure of carbon dioxide (PaCO_2_ ) of 47 mmHg, pH of 7.5, and oxygen saturation of 95%.

Two days before surgery, the patient was transferred to the intensive care unit (ICU) for optimization and she was ventilated with bi-level positive airway pressure (BiPAP) intermittently. She was given chest physiotherapy, nebulization with asthalin, steam inhalation, incentive spirometry, and was taught deep breathing exercises. Bilateral venous Doppler was done to rule out deep vein thrombosis, and a sequential compression device was used for prophylaxis of the same. A 7-Fr triple lumen catheter was secured in the right internal jugular vein under local anesthesia in the ICU.

On the morning of the surgery, the patient's blood sugar level was 178 mg/dL. After premedication with pantoprazole 40 mg and mosapride 10 mg, the patient was transferred to the operating room on her large-sized ICU bed. All equipments required in the case of difficult intubation were kept ready. After attaching monitors, such as, pulse oximeter, cardioscope, and noninvasive blood pressure (large size cuff) monitor, the patient was premedicated with fentanyl 1 µg/kg i.v., midazolam 0.03 mg/kg i.v., and glycopyrrolate 0.2 mg i.v. The patient was induced with propofol 2 mg/kg i.v. We were able to mask ventilate the patient adequately, so intermediate acting muscle relaxant atracurium 0.5 mg/kg i.v. was given. On direct laryngoscopy with stubby handle and Macintosh blade, Cormack Lehane view II was found and we were able to intubate the patient with a 7.5-mm cuffed flexometallic *endotracheal tube*. Proper tube placement was confirmed with end-tidal carbon dioxide (ETCO _2_) and bilateral auscultation of chest being equal and clear. A radial artery catheter of 20 G was placed in the left hand and invasive blood pressure monitoring was done. The eyes were taped shut and padded. The patient was made to lie in a prone position; taking care that the bolsters were placed far enough to prevent abdominal compression. All pressure points were padded with cotton rolls. Her head was placed on a gel head support avoiding eye compression.

Anesthesia was maintained with O _2_ :N _2_O::50:50, sevoflurane 1%–1.5%, fentanyl 1 µg/kg/h i.v., and atracurium 0.2 mg/kg/h i.v. The patient was ventilated with volume control mode with a tidal volume of 550 mL at 16 breaths per min with peak airway pressure of 34 cm H _2_ O with a positive end-expiratory pressure (PEEP) of 6–8 cm of H _2_ O. Initial reading of ETCO _2_ was 36–38 mmHg, which was constantly monitored to diagnose any embolism. She maintained SpO _2_ of 95%–98% and central venous pressure (CVP) around 10–12 cm H _2_ O. Her arterial blood gas analysis, CVP, urine outflow, blood glucose, and temperature were continuously monitored during the surgery. Intraoperative arterial blood gas revealed PaO _2_ of 85 mmHg, PaCO _2_ of 47mmHg, pH of 7.35, HCO _3_ 24, and oxygen saturation of 96%. PaO _2_ /FiO _2_ was 170, PAO _2_ of 297, and the alveolar arterial (A-a) gradient of 212.7. There was an unexplained drop in the blood pressure an hour after incision, which responded to dopamine infusion of 5µg/kg/h. Operative procedure lasted for 3.5 h. Intraoperative course was uneventful. She received diclofenac 75 mg i.v. for postoperative pain relief. At the end of the surgery, neuromuscular blockade was not reversed and she was transferred to the ICU for a gradual weaning. In the ICU, we ventilated her with a 10 cm H _2_ O PEEP and continued respiratory physiotherapy. She was extubated on the second postoperative day and was given BiPAP ventilation till the third postop day. She was transferred back to the ward on the postop day 4 after she had recovered spontaneous breathing and sufficient muscle strength. Further postop period was uneventful and she went home on postop day 7.

## DISCUSSION

Obesity is associated with diseases of many organ systems. Systemic hypertension, pulmonary hypertension, left or right ventricular deficiency, ishemic heart disease, and similar cardiac problems, dyspnea, orthopnea, and similar respiratory signs and problems should be assessed and airway should be paid attention to in the preoperative evaluation of morbidly obese patients.[3] Our patient had diabetes mellitus and hypertension, but her arterial blood pressure and blood glucose level were in normal levels with medical treatment. A baseline arterial blood gas measurement helps to evaluate carbon dioxide retention and provides guidelines for perioperative oxygen administration and weaning from postoperative ventilation.[4] Because of limited effort capacity and respiratory distress in supine position, in most of the obese patients a preoperative optimization in ICU using BiPAP ventilation and respiratory physiotherapy may be beneficial. BiPAP combines pressure support ventilation and PEEP via a nasal mask and allows alveolar recruitment during inspiration and prevents expiratory alveolar collapse. Before the induction of anesthesia in patients with respiratory compromise secondary to factors that reduce functional residual capacity, noninvasive BiPAP in combination with supplemental oxygen may be indicated to provide adequate oxyhemoglobin saturation.

Peripheral and central venous access and arterial cannulation sites should be evaluated during the preoperative examination, and the possibility of invasive monitoring should be discussed with the patient.[4,5] Invasive arterial monitoring should be used for the morbidly obese with cardiopulmonary disease and for those with inappropriate noninvasive blood pressure cuff.[6] Central venous catheterization should be used in patients who have cardiopulmonary disease and have problematic peripheral venous access.[6] We placed an arterial and central venous catheter to measure invasive arterial blood pressure and to provide safe venous access.

During the premedication of these patients' anxiolysis, analgesia and prophylaxis against aspiration pneumonia should be administered.[4] Oral benzodiazepines are suitable for anxiolysis and sedation because they cause little or no respiratory depression. Midazolam i.v. can also be titrated in small doses for anxiolysis during the immediate preoperative period. H _2_ -receptor antagonists and proton pump inhibitors reduce aspiration risk by reducing gastric volume and acidity.[4] We also gave mosapride to prevent aspiration and postoperative nausea and vomiting.

The possibility of a difficult intubation should be anticipated.[4,7] Brodsky *et al*.[8] used a logistic regression model to quantify the relationship between the ease of intubation and patient characteristics. They predicted that odds of a problematic intubation in a particular patient with a neck circumference 1 cm larger than that of another patient are 1.13 times. Therefore, the probability of a problematic intubation was approximately 5% with a 40 cm neck circumference compared with a 35% probability at 60 cm neck circumference.[8] In our case, although the patient had 42 cm neck circumference and the other difficult intubation criteria, we did not encounter any difficulties in intubation.

Morbidly obese patients carry more problems when immobilized. The most important problems being chest infection and deep vein thrombosis. Due to severe low back pain, our patient was confined to bed for almost 4 months. Hence, we did a venous Doppler to rule out *deep vein thrombosis* and also gave prophylaxis for it.

The prone position has a key role in posterior approach in spinal surgery. Ophthalmic complications, such as edema and temporary and permanent acute vision loss have been reported.[9,10] It is still debatable if reduction of intraocular perfusion pressure is due to raised intraocular pressure or due to all reasons of reduction of systemic mean arterial pressure. In the postoperative period, hypoventilation and hypoxia with hypercarbia may occur in morbidly obese patients due to the residual influence of general anesthesia drugs, postoperative atelectasis, and postoperative pain. Therefore, tracheal extubation is considered in obese patients when they are fully awake and have recovered from the depressant effects of anesthetic agents. Re-intubation is more difficult and urgent than initial intubation.

For anesthesiologists, problems of airway and its poor accessibility, add to the extra burden. Reports indicate the occurrence of airway obstruction for various reasons, such as mucous plug, blood clot, defective endotracheal tube,[11‐12] and accidental extubation of a patient while in the prone position during spine surgery.[12,13] Cardiac arrest and fibrillation have been reported.[13] Risk factors, as mentioned in the reported case and review, for intraoperative cardiac arrest in patients in the prone position include the following: cardiac abnormalities in patients undergoing major spinal surgery, hypovolemia, air embolism, wound irrigation with hydrogen peroxide, poor positioning, and occluded venous return.

In this report, the prone position added the risk of airway loss, and the effect of positioning of a morbidly obese patient on rigid longitudinal bolsters was an added risk.

This report underlines the importance of preoperative preparation and optimization of the patient before surgery on one hand and the constant vigil for unusual events and the potential hazards surrounding obese patients in this position, resulting in a successful and satisfactory outcome.
